# Beyond mentorship: the promise and perils of sponsorship in health professions education research

**DOI:** 10.1007/s10459-025-10459-y

**Published:** 2025-07-30

**Authors:** Rebekah Cole

**Affiliations:** 1 Department of Military and Emergency Medicine, Uniformed Services University of the Health Sciences, Bethesda, MD USA; 2 Department of Health Professions Education, Uniformed Services University of the Health Sciences, Bethesda, United States

**Keywords:** Sponsorship, Educational research, Career development, Academic advancement, Mentorship

## Abstract

Mentorship has long been regarded as foundational for academic advancement. However, the potentially transformative role of sponsorship remains underexplored in health professions education (HPE) research. Sponsorship, the proactive leveraging of influence to advocate for others, can offer critical support to early-career researchers. Yet, while sponsorship has clear benefits, it is neither universally necessary, nor free of potential challenges. This article offers a critical examination of sponsorship in HPE research, discussing its advantages, complexities, and potential risks. Drawing from social capital theory and identity frameworks, it explores how sponsorship can both promote and inhibit equity, and it outlines practical considerations for cultivating sponsorship practices that are ethical, inclusive, and sustainable.

## Introduction

Mentorship is widely recognized as a cornerstone of professional development in academia (Sambunjak et al., 2006). Trainees and junior faculty are encouraged to seek out mentors for guidance on research, teaching, and career navigation. However, sponsorship is an often overlooked, but equally vital component of academic success. Unlike mentorship, which emphasizes guidance and support, sponsorship involves active advocacy, opportunity brokering, and direct promotion of a junior faculty member’s career (Ibarra et al., 2010).

Health professions education (HPE) research occupies a unique space in the academic landscape. It is often interdisciplinary, underfunded, and sometimes marginalized within traditional academic hierarchies (Schwartz et al., 2024). As a result, early-career researchers in this field must navigate not only intellectual challenges, but also institutional biases and unclear promotion pathways. Sponsorship helps to mitigate these challenges as it increases access to research collaborations, high-impact authorship opportunities, leadership roles, and presentation opportunities (Williams et al., 2023). It likewise helps early career educational researchers build the academic capital necessary for promotion and tenure (Bourdieu, 1986).

While well-established in leadership and organizational literature, sponsorship has yet to be fully integrated into the culture of health professions education (HPE) research. This gap is particularly concerning given the critical role educational research plays in advancing the health professions. Educational research drives innovation in curriculum design, teaching methodologies, assessment practices, and systems-based learning (Frenk et al., 2010). Without a strong base of rigorous education scholarship, efforts to improve clinical training and patient outcomes may be futile. In addition, because sponsorship does not exist without potential relationship challenges and it is not universally accessible, it holds significant ethical and professional implications for the field of HPE, which have not yet been fully explored in the professional literature.

To close these knowledge gaps, this article examines the potential, limitations, and ethical complexities of sponsorship in HPE research. It considers sponsorship within broader theories of social capital (Bourdieu, 1986) and identity formation (Gee, 2000) in order to explore how sponsorship may simultaneously create opportunity and reinforce exclusionary norms. Overall, a balanced perspective is needed to ensure that HPE researchers can equitably gain support needed to advance their careers.

### Sponsorship versus mentorship: distinct roles and responsibilities

While mentorship and sponsorship are often assumed to be intertwined, they serve distinct purposes. Mentorship provides foundational support: a mentor may review manuscripts, offer feedback on grant proposals, or provide career advice. However, many of these interactions are private and may not lead to tangible career advancement. Sponsorship, in contrast, involves public acts of advocacy. For example, sponsors nominate early career researchers for awards, invite them to co-author papers, recommend them for leadership positions in professional organizations, and provide access to influential professional networks (Sambunjak et al., 2006; DeCastro et al., 2013). While a mentor might help a junior faculty member revise a manuscript, a sponsor introduces them to an editor and recommends the manuscript for fast-track review. In the same way, a mentor might advise the mentee on how to secure grant funding, while a sponsor invites the sponsoree to serve as a co-investigator on a funded grant. These actions elevate a junior researcher to positions of influence and access, a concept aligned with Lave and Wenger’s (1991) theory of legitimate peripheral participation.

Both sponsors and their sponsorees carry responsibilities within the relationship. First, sponsors must consider whose careers they elevate and at what cost, striving to act transparently and equitably. Sponsorees should likewise be aware of potential obligations or dependencies and navigate these relationships with clarity and professionalism (Williams et al., 2023). Institutions can play a critical role by offering faculty development initiatives to educate senior faculty about ethical and inclusive sponsorship practices (Haas et al., 2023) and openly discussing sponsorship as an integral aspect of university culture.

### Types of sponsorship

Sponsorship can take multiple forms including: (1) formal sponsorship, which involves nominating sponsorees for leadership positions or awards (DeCastro et al., 2013); (2) informal sponsorship, such as speaking favorably about a sponsoree in hiring or grant review contexts; and (3) symbolic sponsorship, which includes publicly praising sponsorees in influential settings, which can elevate visibility and promotion opportunities (Ibarra et al., 2010).

These different types of sponsorship align with Bourdieu’s (1986) concept of social capital, in which access to networks and recognition become critical forms of professional currency. Sponsorship practices also intersect with identity formation theory, which explains how professional identity is often shaped by the institution. Early career HPE researchers being sponsored may be chosen based on perceived alignment with institutional values or norms, potentially encouraging them to shed their individualities and conform to the values, ideas, and norms of the institution (Gee, 2000).

However, alongside its benefits, sponsorship has its challenges. While it can create pathways to opportunity, sponsorship can also reinforce existing hierarchies if sponsors tend to favor early career HPE researchers who resemble themselves or reflect established institutional norms (Ibarra et al., 2010). Consequently, HPE scholars whose research challenges prevailing paradigms or whose personal or professional identities differ from the dominant culture might be less likely to receive sponsorship, potentially stifling innovation and diversity in HPE research.

### Is sponsorship in health professions education research really necessary?

Sponsorship is often portrayed as essential for career success in competitive academic environments (Williams et al., 2023), as evidence suggests that sponsorship can accelerate promotion, increase visibility, and open doors to networking opportunities (Schwartz et al., 2024). However, not all successful HPE researchers have had sponsors, and sponsorship is not the only route to advancement. HPE researchers can also succeed through resilience, self-advocacy, or other personal or professional strengths (Bourdieu, 1986; Cook et al., 2008). Therefore, while sponsorship can be a powerful accelerator, it is not strictly necessary for all. However, it is important to note that the absence of sponsorship may leave some HPE researchers at a disadvantage in academic and professional systems where visibility and connections are critical for advancement. Thus, sponsorship might be classified as highly advantageous, but not universally required.

### Potential risks of sponsorship

Although sponsorship is often advantageous for HPE researchers, it can also carry significant risks. For example, toxic sponsorship could potentially occur when sponsors expect loyalty, request labor without reciprocity or compensation, or leverage their influence for personal or political gain (DeCastro et al., 2013). This dynamic can be particularly damaging for early-career HPE researchers who may feel obligated to comply due to the power imbalance in these relationships. Finally, sponsorship can potentially create vulnerable dependency. For example, if a sponsor loses power, retires, or shifts professional focus, sponsored individuals may find their own reputations or career opportunities hindered. Such dependencies highlight the need for diversification of networks and caution in relying too heavily on a single sponsor (Sambunjak et al., 2006).

### Inequitable access to sponsorship

In this reflection on sponsorship, it is important to note that access to sponsorship is not equally distributed. Scholars from historically marginalized groups may face barriers in finding sponsors because sponsorship may emerge within informal networks shaped by shared demographic characteristics (Ibarra et al., 2010; Williams et al., 2023). Without intentional interventions, sponsorship risks creating a two-tiered system where some privileged faculty might fast-track their careers, while others remain unsupported. Bourdieu’s (1986) theory of social capital underscores that advancement in academia depends not only on merit, but also on access to networks and recognition. As a result, institutions that fail to address disparities in sponsorship may perpetuate these systemic inequities.

### Institutional implications and recommendations

Recognizing the complex nature of sponsorship calls for intentional institutional strategies. Table 1 outlines institutional recommendations that align with calls to transform health professions education into a system that is interdependent, equitable, and responsive to societal needs (DeCastro et al., 2013; Frenk et al., 2010; Haas et al., 2023; Schwartz et al., 2024).

**Table 1:** Institutional recommendations for ethical and equitable sponsorship

Institutional strategy	Description/rationale
Train senior faculty on ethical sponsorship practices	Equip faculty with knowledge and skills to sponsor responsibly, avoiding bias or exploitative dynamics.
Establish formal recognition systems for sponsors	Integrate sponsorship into definitions of academic citizenship and criteria for career advancement to incentivize ethical sponsorship.
Create accessible pathways for early career HPE faculty to seek sponsorship	Reduce reliance on informal networks by providing clear processes for finding sponsors, thus promoting equity.
Encourage multiple sponsors for early career faculty	Reduce dependency on a single sponsor and expand networks for diverse opportunities and support.
Provide oversight and accountability for sponsors	Ensure sponsorship relationships remain ethical, transparent, and beneficial to sponsorees.
Embed sponsorship into academic culture as a valued, collective practice	Promote sponsorship as a deliberate, institutionally supported strategy to foster an inclusive and evidence-informed HPE research environment.

## Conclusion

Sponsorship offers potential benefits for advancing careers in HPE research. However, sponsorship is not universally necessary, and may carry risks for sponsorees of inequity, dependency, and the reinforcement of existing hierarchies. Institutions, faculty development programs, and senior scholars all have a role in ensuring that sponsorship contributes to a more inclusive and academic culture.

## Data Availability

No datasets were generated or analysed during the current study.

## Declarations

The opinions and assertions expressed herein are those of the author and do not reflect the official policy or position of the Uniformed Services University of the Health Sciences or the Department of Defense.

ChatGPT was used to copy edit this manuscript.
